# Local delivery of simvastatin maintains tooth anchorage during mechanical tooth moving via anti‐inflammation property and AMPK/MAPK/NF‐kB inhibition

**DOI:** 10.1111/jcmm.16058

**Published:** 2020-12-12

**Authors:** Lianyi Xu, Xiaojuan Sun, Guangxun Zhu, Jing Mao, Babak Baban, Xu Qin

**Affiliations:** ^1^ Department of Stomatology Tongji Hospital Tongji Medical College Huazhong University of Science and Technology Wuhan China; ^2^ Department of Oral and Maxillofacial Surgery General Hospital Ningxia Medical University Yinchuan China; ^3^ Department of Oral Biology and Diagnostic Sciences The Dental College of Georgia Augusta University Augusta GA USA

**Keywords:** AMPK, animal study, MAPK, mechanical stress, periodontal ligament cells, simvastatin

## Abstract

Simvastatin (SMV) could increase tooth anchorage during orthodontic tooth movement (OTM). However, previous studies on its bone‐specific anabolic and anti‐inflammation properties were based on static in vitro and in vivo conditions. AMPK is a stress‐activated kinase that protects tissue against serious damage from overloading inflammation. Rat periodontal ligament cells (PDLCs) were subjected to a serial of SMV concentrations to investigate the optimization that promoted osteogenic differentiation. The PDLCs in static and/or tensile culturing conditions then received the proper concentration SMV. Related factors expression was measured by the protein array, real‐time PCR and Western blot. The 0.05UM SMV triggered osteogenic differentiation of PDLCs. The inhibition of AMPK activation through a pharmacological approach (Compound C) caused dramatic decrease in osteogenic/angiogenic gene expression and significant increase in inflammatory NF‐κB phosphorylation. In contrast, pharmacological activation of AMPK by AICAR significantly inhibited inflammatory factors expression and activated ERK1/2, P38 MAPK phosphorylation. Moreover, AMPK activation induced by SMV delivery significantly attenuated the osteoclastogenesis and decreased the expression of pro‐inflammatory TNF‐α and NF‐κB in a rodent model of OTM. The current studies suggested that SMV could intrigue intrinsic activation of AMPK in PDLCs that promote attenuate the inflammation which occurred under tensile irritation through AMPK/MAPK/NF‐kB Inhibition.

## 1 INTRODUCTION

Dentofacial deformity brings a lot of physical and psychological distress to the patients.^1^ Orthodontic tooth movement (OTM) is the main strategy for solving the problem by applying fix/removable mechanical means to adjust the teeth arrangement that involves a biomechanical adaptation of the alveolar process and supported periodontium.^2^ The introduction of biological therapy to assist this mechanically induced physical movement raises great attention for shortening the treatment cycle and reducing the patients’ incompliance caused by pain and discomfort, by either accelerating local movement rate or precisely increasing tooth anchorage in the alveolar.^3^ Existing biological therapy includes chemical methods (cytokines, hormone, drugs, growth factors and other biological substances) and gene therapy, among which the most potential translational approach is the locally administrated meditations.^4^

Simvastatin (SMV), as the HMG‐CoA reductase inhibitor, is a cholesterol‐lowering statin drug.^5^ Recently, its protective bone anabolic and anti‐resorptive properties have been unveiled and lead to a wide application in large‐size bone defect regeneration, therapeutic management of osteoporosis and periodontitis correction.^6^, ^7^, ^8^ According to clinical trials observation, SMV intake was beneficial for chronic periodontitis control for its function in inflammation limitation and periodontal tissues repair.^9^, ^10^, ^11^ Animal studies have shown that SMV when administered systemically resulted in increased tooth anchorage and reduced root resorption.^3^, ^12^ To be mentioned, the pocket injection of 1.2% SMV gel decreased space re‐opening after orthodontic space closure in human anterior teeth.^13^ We thus assume a bone anabolic property of SMV on periodontal tissue under a biomechanically induced inflammatory condition.

Adenylate‐activated protein kinase (AMPK) is a silk and threonine‐protein kinase which mainly coordinates the metabolism of energy.^14^, ^15^ Lately, researchers discovered its roles in autophagy inducement, inflammation control, cancer metastasis and autoimmune inhibition.^16^, ^17^, ^18^, ^19^, ^20^, ^21^, ^22^ Our group also found that AMPK‐α1 knockout (AMPK‐α1^‐/‐^) mice presented larger inflammatory periodontal bone defects and expressed higher levels of inflammatory cytokines than wild‐type (WT) mice.^23^ Interestingly, OTM is a biomechanical process accompanied by tension side bone regeneration and pressure side bone resorption, which indicates the AMPK activation in the inflammation‐mediated osteogenesis under biomechanical irritation.^24^

Current in vitro observations grounded on the static‐culturing periodontal ligament cells (PDLCs), which led to big inconsistency from the actual stress state of the PDLCs during OTM. In this study, we focused on the anti‐inflammation effect of SMV on tensile side of the orthodontic moving tooth in a rodent model. An in vitro dynamic culturing system was also applied to mimic the stressing condition in OTM to explore the potential mechanism concerning the AMPK regulation.

## 2 MATERIALS AND METHODS

### 2.1 Animal for OTM model

Twelve‐week‐old male Sprague Dawley (SD) rats of 250 g ± 15 g were obtained from the Tongji Medical College Animal Center. All animal procedures were approved by the Animal Care and Experiment Committee of Tongji Hospital, Tongji medical college, Huazhong University of Science and Technology. Animals were housed in specific pathogen‐free (SPF) condition at an ambient 24‐26°C temperature, 55%‐60% humidity under a 12:12‐hour light/dark cycle. Adequate measures were implemented to minimize pain or discomfort of animals during all procedures. The study compliance with the ARRIVE guidelines.

### 2.2 Acquisition and cultivation of human PDLCs

Ten human healthy pre‐molars were collected from four patients (18‐25 years old) undergoing orthodontic treatment in the Department of Stomatology, Tongji Hospital. Informed consent was signed by all patients according to the Tongji Hospital, Huazhong University of Science and Technology Institutional Review Board Approval (IRB ID: 20140401) under the principles of the Declaration of Helsinki. After phosphate buffered saline (PBS) washing, the periodontal ligament tissue was dissected from the mid‐third portion of the roots using a sterile NO. 11 blade (Golden Circle Medical Supplies Co., Ltd.). The tissue explants were carefully placed on the bottom of a six‐well culture plates and covered by a sterilized glass before adding Dulbecco's modified Eagle's medium (DMEM, Gibco BRL) supplemented with 10% foetal bovine serum (FBS, Gibco Biocult Co), 100 U/mL penicillin and 100 mg/L streptomycin (Invitrogen Life Technologies).^25^ The explants were kept in a 5% CO_2_ incubator at 37°C. Cells at passage 3‐5 were adopted for the following tests.^26^

### 2.3 The optimization of SMV concentration

Periodontal ligament cells were seeded into 24‐well plates at a density of 4.0 × 10^4^ cells/mL. One day later, six concentrations of SMV (0, 0.01, 0.05, 0.1, 0.5 and 1UM) (Sigma‐Aldrich) in supplemented DMEM were offered. The cell numbers in each hole were evaluated on days 1, 4, 7 and 10 (Multisizer, Beckman Coulter Inc). PDLCs were seeded in 6‐well plates at a density of 5 × 10^4^ cells/well and cultured in the six concentrations prepared in osteogenic culture medium (supplemented DMEM with 50 mg/mL ascorbic acid, 10 mmol/L b‐glycerophosphate and 10^−8^ mol/L dexamethasone, Sigma). After 7‐ and 14‐day inducement, ALP staining (Beyotime Biotech, Jiangsu, China) and semi‐quantitative analysis (Sigma) were performed. The total protein content was determined using a protein assay kit (Bio‐Rad). The osteogenic gene expression of RUNX2, VEGF, OPN and OCN was measured by RT‐PCR on days 1 and 7. Finally, after 4 weeks inducement, fixed PDLCs were marked by the Alizarin Red staining ^27^ and the Von Kossa staining.^28^ Osteogenic medium (0UM SMV) was used as control. All experiments were performed in triplicate.

### 2.4 Application of tension force system

External mechanical irritation was stimulated with a Flexcell FX‐5K^®^ tension system (Flexcell International Corp.). PDLCs were plated onto six‐well Bioflex plates (Flexcell International Corp.) at a density of 1 × 10^5^ cells/well. When the culture reached 80%‐90% confluence, they were stretched with 10% equibiaxial strain at 0.1 Hz for 24 hours.^29^ PDLCs were assigned to six groups as:

- C‐CON: No tension and no SMV (osteogenic culture medium only);
- C‐SMV: No tension + 0.05UM SMV;
- T‐CON: Tension only;
- T‐SMV: Tension + 0.05UM SMV (given 10 minutes before the stress loading);
- T‐Compound C: Tension + 0.05UM SMV + 1UM Compound C (Compound C were given 10 minutes before the SMV dosing);
- T‐AICAR: Tension + 0.05UM SMV + 1UM Compound C + 1MM AICAR (AICAR were given 10 minutes before the Compound C dosing).

### 2.5 Protein array

The human protein membrane array (QAH‐PDD‐1, Ray Biotech) simultaneously profiles 20 proteins were in quadruplicate, and the supernatant of group i, ii, iii and iv was pulled together for measurement.^30^ The cytokine array membranes were blocked for 30 minutes and for 2 hours in the collected supernatants. After washing, the membranes were incubated with biotin‐conjugated antibodies (1:250 dilution, 1 mL per array membrane) at room temperature for 2 hours and washed again. The horseradish peroxidase‐conjugated streptavidin solution (1:1000, 2 mL) was added and incubated for 2 hours followed by a third wash step. Proteins were detected by the enhanced chemiluminescence, and the membranes were exposed to X‐ray films.

### 2.6 Real‐time Quantitative PCR

The total cellular RNA was extracted using the TRIzol one‐step method (Invitrogen Life Technologies) and reverse‐transcribed to cDNA using a cDNA Synthesis kit (TaKaRa Bio, Inc, Otsu, Shiga, Japan). Primers were synthesized commercially (Shengong Co. Ltd) and were shown as follows: GAPDH, 5′‐ GACCTGACCTGCCGTCTA ‐3′ and 5′‐AGGAGTGGGTGTCGCTGT‐3′; OCN, 5′‐ CTCACACTCCTCGCCCTATT ‐3′ and 5′ ‐GCCTGGGTCTCTTCACTACCT ‐3′; OPN, 5′‐ ACTGATTTTCCCACGGACCT ‐3′ and 5′‐ CATTCAACTCCTCGCTTTCC ‐3′; RUNX2, 5′‐ CCATAACCGTCTTCACAAATCC ‐3′ and 5′‐ GCGGGACACCTACTCTCATACT ‐3′; VEGF, 5′‐ AGGGCAGAATCATCACGAAGT ‐3′ and 5′‐ AGGGTCTCGATTGGATGGCA ‐3′; TNF‐α, 5′‐ CCCGACTATCTCGACTTTGC ‐3′ and 5′‐ GGTTGAGGGTGTCTGAAGGA ‐3′; BMP‐2, 5′‐ CAGAAACGAGTGGGAAAACAAC ‐3′ and 5′‐ ATTCGGTGATGGAAACTGCTAT ‐3′; NF‐κB, 5′‐ CGACTATCTCGAGACCTTT ‐3′ and 5′‐ GCCTGGGCTCTCGTCTTCAC ‐3′; IL‐6, 5′‐ GCACCTCA ATTGTTGTTG ‐3′ and 5′‐ AAATAGTGTCCTAACGCTCA ‐3′; AMPK‐α, 5′‐ TCCCTATCTCGTCGAC ‐3′ and 5′‐ CGACCTGCTCGTGGCC ‐3′;. The genes expression was evaluated using a real‐time PCR kit (TaKaRa Bio). GAPDH was used as the housekeeping gene. The 2^−∆∆CT^ method was used to quantify the folded expression level. All tests were repeated in triplicate.

### 2.7 Western blotting analysis

Total proteins of the cells were extracted (RIPA lysis buffer, Sigma) and measured (BCA Protein Assay Kit, Pierce Biotechnology). Protein homogenates were run on SDS‐PAGE gel (Beyotime) and transferred onto a PVDF membrane (Millipore). After 5% milk blocking, the membranes were incubated overnight in primary antibodies: AMPK (1:1000; Beyotime), P‐AMPK (1:1000; Beyotime), p‐p65 (1:2000), and p65 (1:2000), ERK1/2 (1:2000), p‐ERK1/2, p38 MAPK and p‐p38 (Abcam) at 4°C. GAPDH served as the internal reference. After secondary antibodies (Abcam) incubation for 2 hours at 37°C, the membranes were subjected to the enhanced chemiluminescence for signal intensity quantification (Bio‐Rad).

### 2.8 Pre‐treatment of drug injection and Orthodontic model establishment

Four groups of pre‐conditioning were randomly given to all animals: (a) Control group, 70 mL saline once three days via submucosal injection 2 mm under the gingival papilla between the maxillary right first (M1) and second (M2) molar; (b) SMV group, 70 mL 100 mg/kg SMV; (c) Compound C group, 10 mg/kg Compound C was given 10 minutes before SMV administration; and (d) AICAR group, 10 minutes after the Compound C administration, AICAR (30 mg/kg) was given. A total of 3 times dosing were completed before orthodontic appliance fixation.^31^ The orthodontic wire was a titanium‐nickel alloy measuring 0.228 mm in diameter and 14 mm in length.^32^ After intraperitoneal injection of 3.5 mg/100 g pentobarbital, the buccopalatal grooves of the M1 and M2 were enlarged with a diamond bur (NO. 145, Shofu) on the occlusal surfaces to deep enough to fit the spring wire. The site was dried, etched with 65% phosphoric acid (Shofu) for 20 seconds, rinsed with water and dried. The tips were brought together and maintained at a distance of 3 mm by a circular frame to deliver an initial force of 30 g. The springs were seated into the occlusal grooves and bonded with dental resin (Shofu). The frame was removed to activate the spring after bonding. The rats were then allowed to recover from anaesthesia and returned to their cages.

At the end of 1 week active tooth movement, the orthodontic appliances were removed. Animals were euthanasia and perfused with 10% buffered formalin. The maxillae were carefully isolated and trimmed into single blocks together with three right upper molars before storage in 4% neutral formaldehyde solution at 4°C overnight.^33^

### 2.9 Micro‐CT measurement

The alveolar samples were assessed using a micro‐CT system (μCT‐80, Scanco Medical, Bassersdorf, Switzerland) as previously described. The microfocus of the X‐ray source had a spot size 7 mm and maximum voltage 36 kV.^34^ A cylindrical region of interest (ROI) in all the samples mesial to M2 with an axis depth/length of 600 μm (100‐700 μm below the M2 root furcation) and a diameter of 700 μm was selected for measurement of bone volume fraction (BV/TV, %), and the distance between the ROI and the mesial root of M2 was 200 μm.^35^ The amount of tooth movement was assessed on 2‐dimensional (2‐D) sagittal sections taken through the centres of the M1 and M2 (the image plane that showed the most structure of the distobuccal and mesial root) and measured at the interproximal heights of contour between the most mesial point of the M2 crown and the most distal point of the M1 crown.^36^

### 2.10 Histological and histomorphometric assays

All samples were decalcified in 10% EDTA before embedding. The specimens were cut into 5 μm sections and prepared for H&E staining. The histomorphometric analysis was performed under the bright‐field setting on a light microscope (Leica‐microsystems) on six samples per group.^37^ On three randomly selected images (200 × magnification) on the tensile side of the distal root of the first molar per sample, the cell nuclei and Howship's lacunae was calculated (image J software 5.0). Immunohistological staining of TNF‐α and NF‐κB (1:500; Abcam) was performed to locate the protein expression on six samples per group (n = 6). On three randomly selected images (40 × magnification) on the tensile side of the distal root of the first molar per sample, positive cell numbers were calculated.^38^ Finally, the sections were treated with a mixture of a tartaric acid solution and acid phosphatase substrates (Sigma).^28^ The TRAP labelled cells were calculated in the corresponding region of H&E‐stained sections.

### 2.11 Statistical analysis

The experimental data were presented as the mean ± standard derivation. Statistical analysis in this study was performed using one‐way ANOVA and the SNK post hoc test based on the normality of the distribution with the SAS 8.2 statistical software package. Values of *P* < .05, *P* < .01 were considered statistically significant.

## 3 RESULTS

### 3.1 The 0.05UM SMV induced stronger osteogenic differentiation of rat PDLCs in vitro

**Figure 1:**
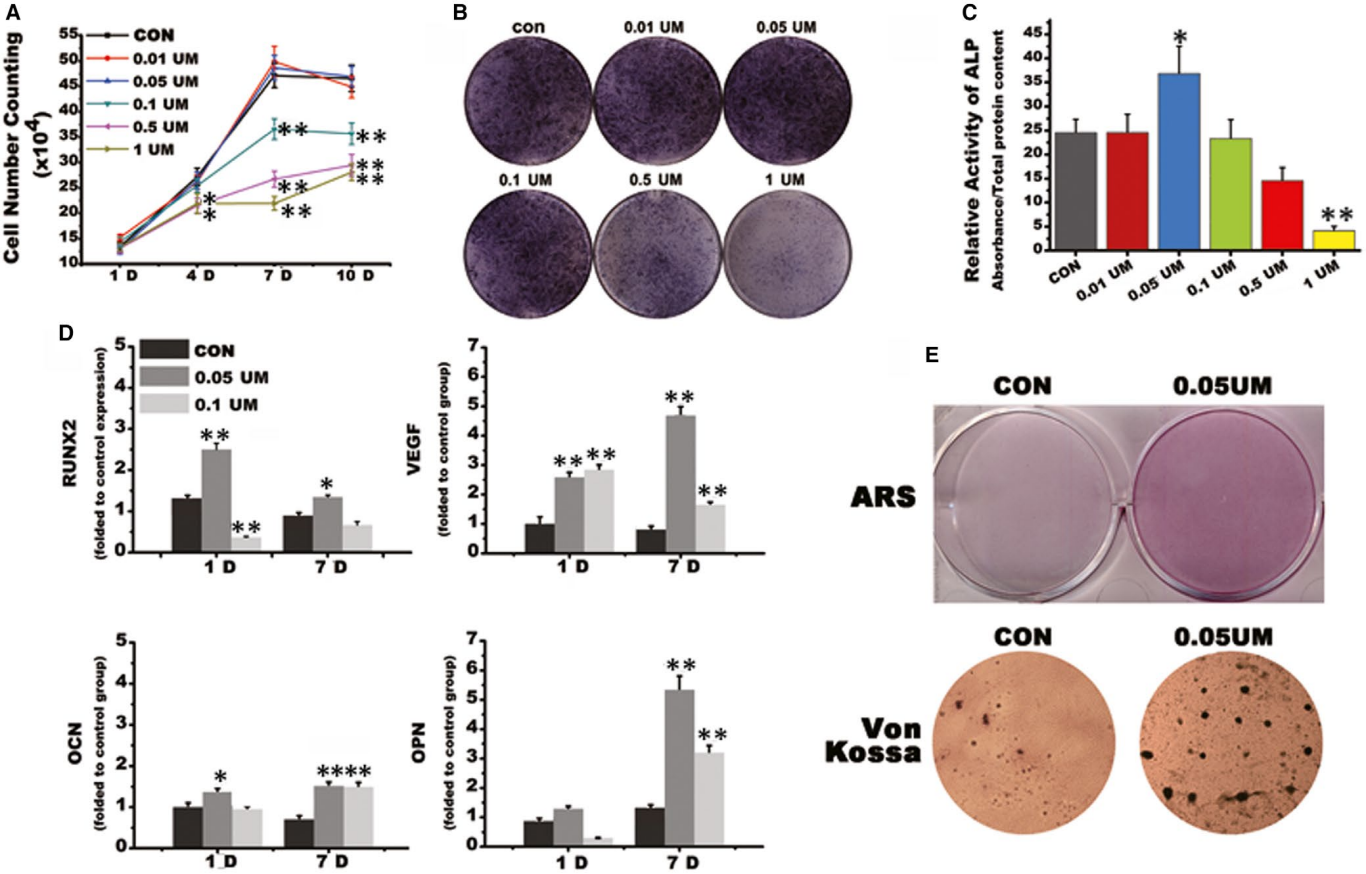
SMV concentration optimization. A, Cell number counting. B, ALP staining on day 7. C, Relative activity of ALP on day 7. D, Real‐time PCR of osteogenic‐related genes. E, Calcified nodule formation (CON for osteogenic induction medium (DX) without SMV; *, *P* < .05, ** *P* < .01 vs CON group)

Human PDLCs were successfully obtained for in vitro tests. To figure out a proper SMV concentration for in vitro tests, we first compared the PDLCs proliferation rate in an increasing concentration gradient of the drug. The cell number gradually increased in all groups as the inducement time prolonged. The proliferation rate of the low SMV concentration groups (0.01 and 0.05UM) was similar to the control group. The proliferation rate of the high SMV concentration groups (0.1, 0.5 and 1UM) slowed from day 4 and reached the platform period after day 7 (Figure 1A). Next, the ALP staining was performed on day 7. For the 0.05UM group, the purple‐red colour stained area reached the maximum. As the SMV concentration increased, the colour of ALP became lighter (Figure 1B). Semi‐quantitative detection of ALP was further performed on day 14. Simply, the ALP activity of 0.05UM group was significantly higher than the control group (*P* < .05), while 1UM group demonstrated the lowest ratio (*P* < .01; Figure 1C).

We then selected two concentrations of 0.05 and 0.1UM for further assessments. The two‐step RT‐PCR showed that OCN expressions were similar among two testing groups, while the 0.05UM group stimulated higher expressions of RUNX2 (1‐day induction) and OPN (7‐day induction) (*, *P* < .05; **, *P* < .01). The expression of angiogenic factor VEGF in both testing groups significantly increased on the 1st day and reached the highest level in the 0.05UM group on the 7th day (**, *P* < .01). The change of gene expression level suggested that 0.05UM SMV induced the strongest osteogenic and angiogenic differentiation in PDLCs (Figure 1D). Finally, we tested the calcified nodule formation in control and 0.05UM SMV groups. Both Alizarin Red and Von Kossa staining showed that the 0.05UM SMV induced more calcified nodules in larger size than the control group (Figure 1E).

### 3.2 SMV promoted osteogenesis on the tensile side of periodontium during OTM via anti‐inflammatory effect

**Figure 2:**
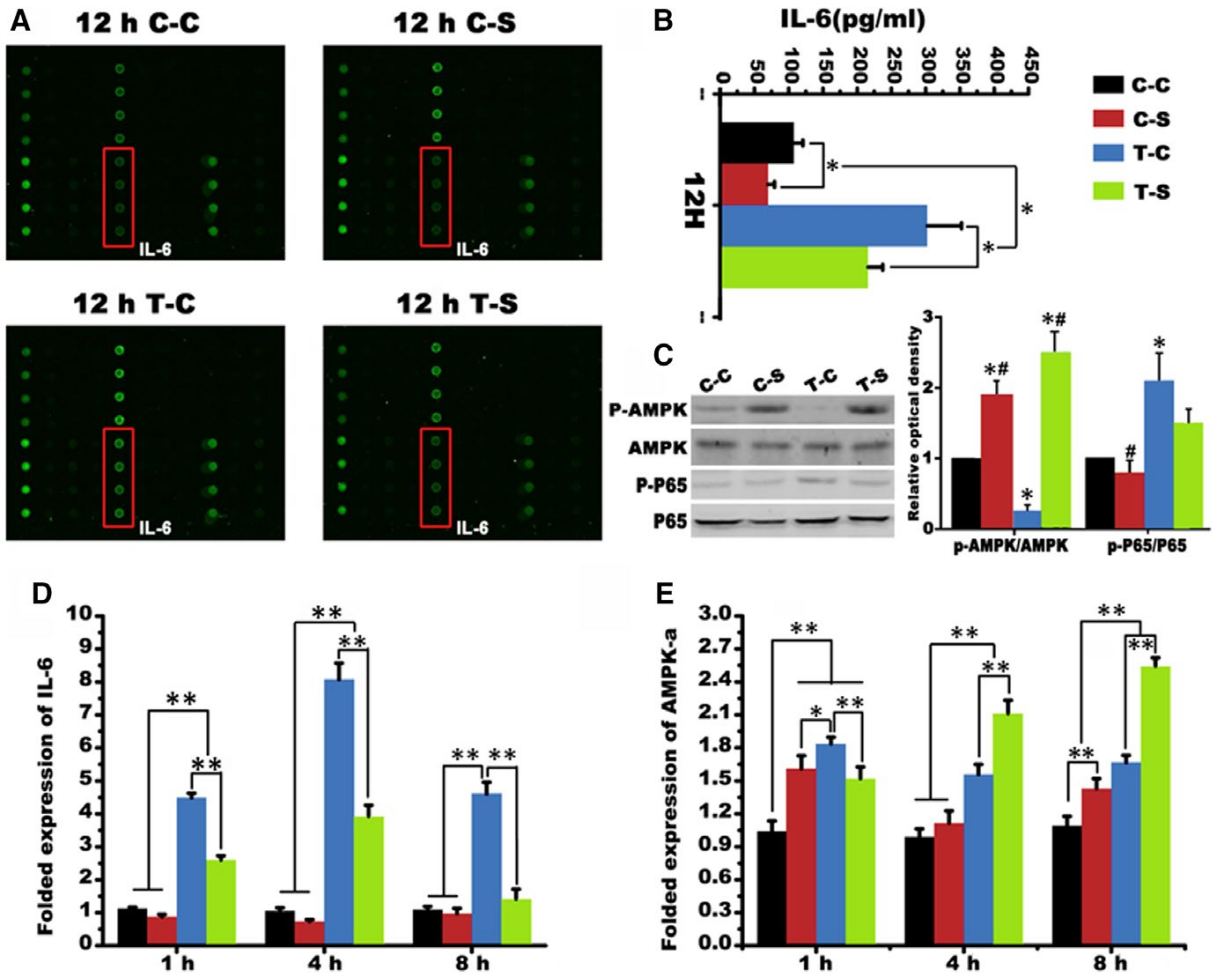
Protein secretion of PDLCs. A, Human cytokine antibody array screening of supernatant derived from co‐stimulated PDLCs. B, IL‐6 expression in supernatants derived from indicated treatment. C, The Western blot of AMPK and P65 and the folded relative optical dentistry of p‐AMPK/AMPK and p‐P65/ P65 (*, *P* < .05, ** *P* < .01 vs C‐C group; # *P* < .05, ##, *P* < .01 vs C‐S group; @, *P* < .05, @@, *P* < .01 vs T‐C group). D, Folded expression of IL‐6 indicated by Real‐time PCR. E, Folded expression of AMPK‐ɑ mRNA (*, *P* < .05, ** *P* < .01)

SMV shows protective bone anabolic and anti‐resorptive properties.^6^, ^11^ We here focused on identifying the intrinsic factor(s) responsible for the therapeutic effects of SMV in increasing tooth anchorage. We took advantage of protein array technology and scanned 20 pro‐ and anti‐inflammatory factors in the co‐stimuli of tension and SMV‐sensitized PDLCs. We found that IL‐6 was the most pronounced cytokine induced by PDLCs under the co‐stimuli (Figure 2A,B). To detect the potential effect of AMPK during SMV‐induced anti‐inflammation, we subsequently measured the expression levels of p‐AMPK/AMPK and p‐P65/P65 (NF‐κB) in the cells. Our data demonstrated that increased AMPK was negatively connected with IL‐6 secretion (Figure 2C). Also, we confirmed that IL‐6 mRNA were correspondingly reduced after indicated treatment and AMPK mRNA levels were correspondingly elevated by the co‐stimuli (Figure 2D,E).

**Figure 3:**
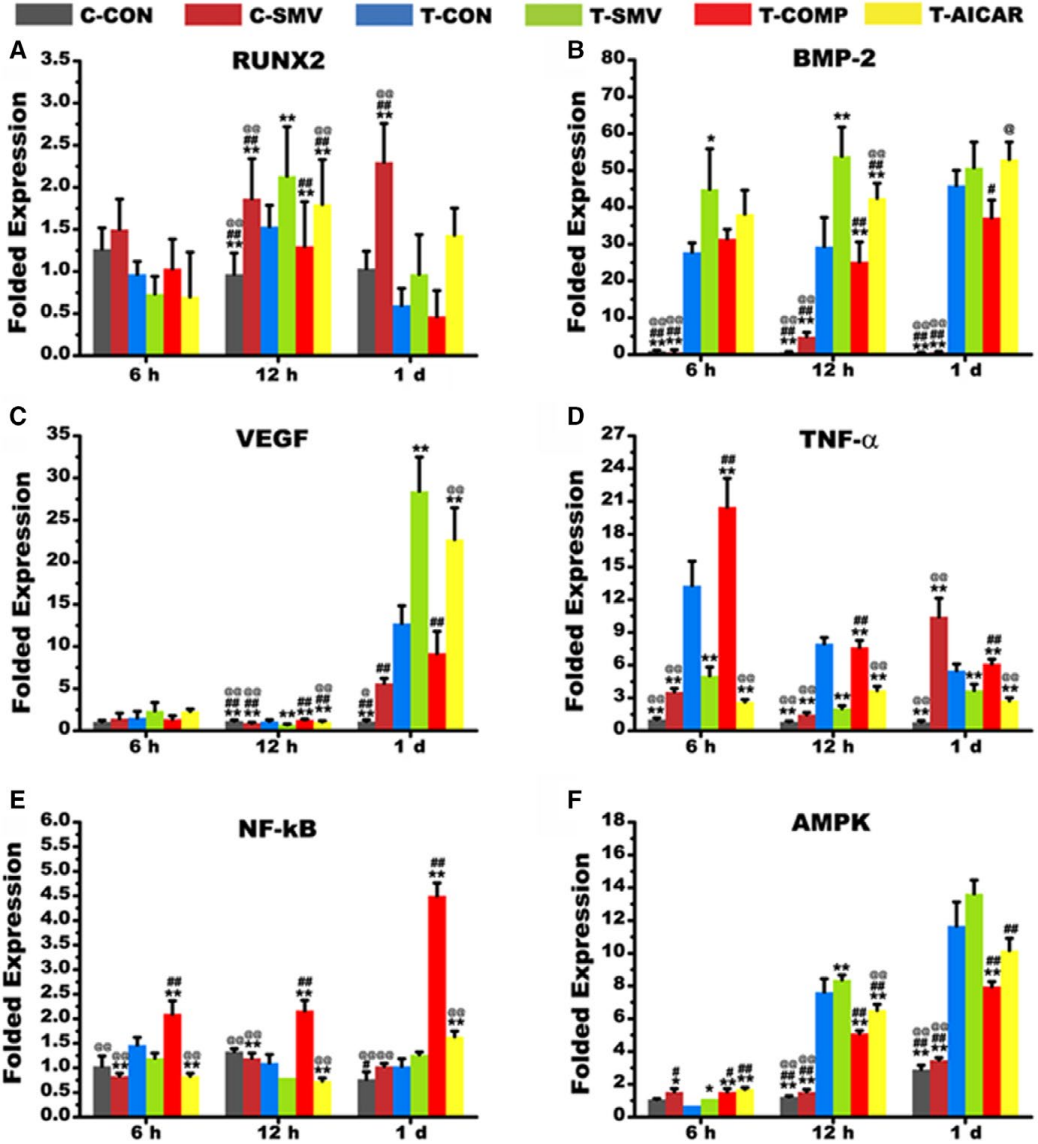
Real‐time PCR detection after 6‐h, 12‐h and 1‐d inducement. A, Folded expression of RUNX2. B, Folded expression of BMP‐2. C, Folded expression of VEGF. D, Folded expression of TNF‐ɑ. E, Folded expression of NF‐κB. F, Folded expression of AMPK (*, *P* < .05, ** *P* < .01 vs T‐CON group; # *P* < .05, ##, *P* < .01 vs T‐SMV group; @, *P* < .05, @@, *P* < .01 vs T‐COMP group)

The intervention of AMPK signalling was analysed by the administration of the AMPK inhibitor, Compound C and the AMPK activator, AICAR. RUNX2, BMP‐2 and VEGF are important factors during osteogenesis. For BMP‐2 and VEGF, the mechanical tension significantly increased their expression compared to the static culture condition (*P* < .01). Unlike VEGF expression, which tended to increase over time, BMP‐2 expression remained high within 24 hours. For RUNX2, mechanical stress did not induce obvious augmentation of its expression level as did in BMP‐2 and VEGF. However, the addition of SMV significantly lifted the expression level in all the three osteogenic‐related genes (*P* < .05; Figure 3A‐C). For pro‐inflammatory factors, the SMV administration increased its expression under a static environment (*P* < .05). This change was reversed under mechanical culturing condition. The pre‐conditioning of Compound C and AICAR further proved this trend (*P* < .01; Figure 3D). For NF‐κB, the inhibition of AMPK significantly elevated its expression in all the time‐points (*P* < .01). This change was corrected after the administration of AMPK activator. However, the tensile culture condition did not show statistic influence on RUNX2 expression (Figure 3E). On the other hand, the expression level of AMPK significantly increased in tensile condition at 12 hours and 1 day. And SMV collaboratively increased the expression level at 6 hours (*, *P* < .05) and 12 hours (**, *P* < .01). This alteration could be inhibited by Compound C (Figure 3F). Meanwhile, there was no significant difference in the expression of AMPK‐α (*P* > .05).

### 3.3 SMV promoted osteogenesis on the tensile side of periodontium during OTM via AMPK/MAPK/ NF‐ΚB inhibition

**Figure 4:**
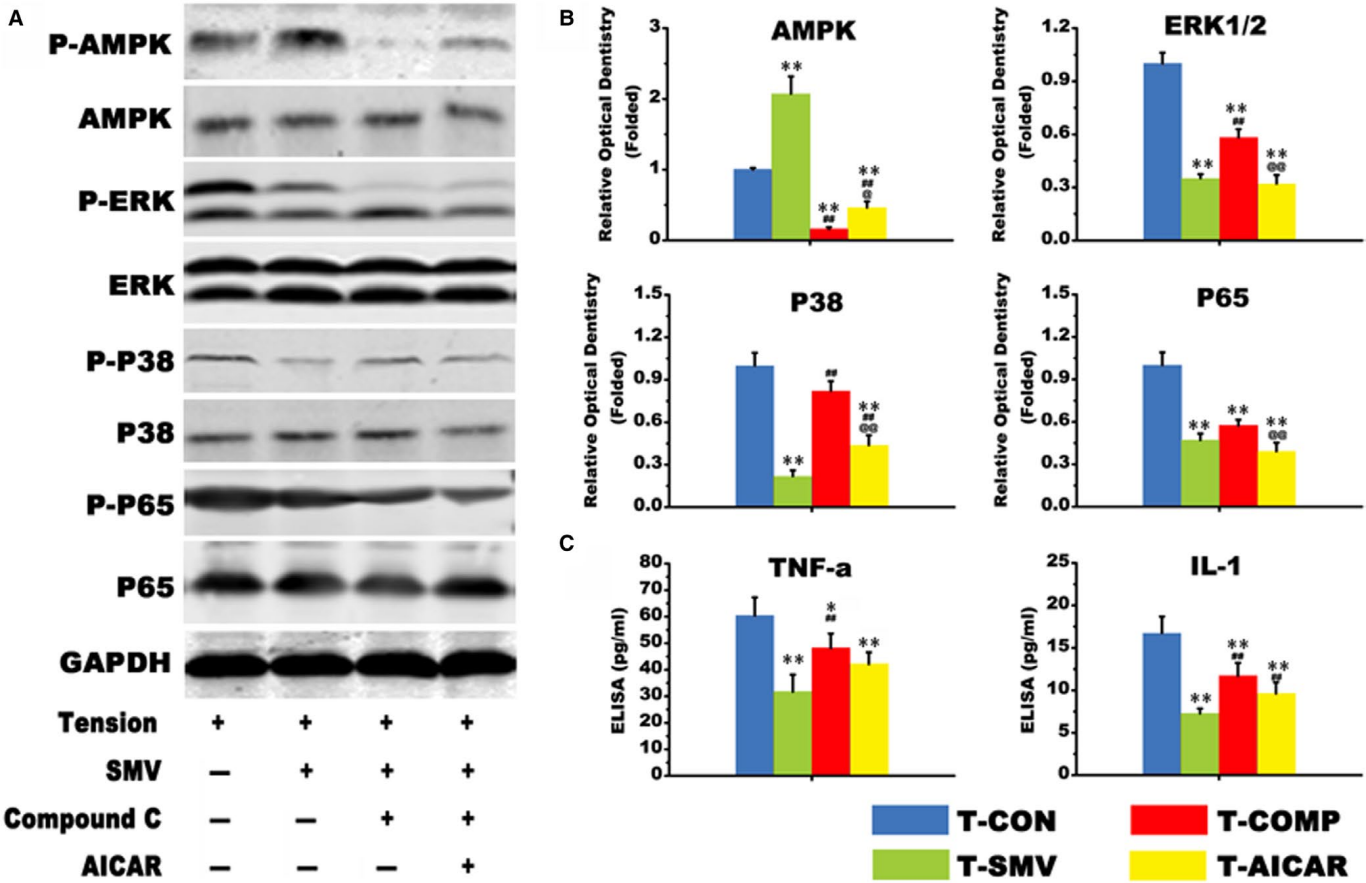
Western blot. A, The protein expression of AMPK, ERK1/2, P38, P65 and their phosphorylation levels. B, Relative optical dentistry of p‐AMPK/AMPK, p‐ERK/ERK, p‐P38/P38 and p‐P65/P65 (folded change; *, *P* < .05, ** *P* < .01 vs T‐CON group; # *P* < .05, ##, *P* < .01 vs T‐SMV group; @, *P* < .05, @@, *P* < .01 vs T‐COMP group)

To further clarify the effect of AMPK on SMV stimulated PDLCs in tensile culture condition, we detected the MAPK/NF‐κB signalling by Western blot (Figure 4A). The results showed that 0.05UM SMV treatment down‐regulated the folded expression of p‐ERK, p‐P38 and p‐P65 (##, *P* < .01 vs T‐SMV group) (Figure 4B). This change would be altered by Compound C (@@, *P* < .01 vs T‐COMP group) and further corrected by AICAR, which showed a negative correlation with the p‐AMPK level. Finally, we detected the expressions of TNF‐α and IL‐1 in supernatants derived from indicated treatment by ELISA. The amounts of secreted TNF‐α and IL‐1 shown similar change patterns as the NF‐κB p65 phosphorylation level as induced by the co‐stimuli of tension and SMV (Figure 4C). Specifically, SMV decreased the inflammatory factors level (**, *P* < .01 vs T‐CON group).

### 3.4 Surgical procedures and gross observation

**Figure 5:**
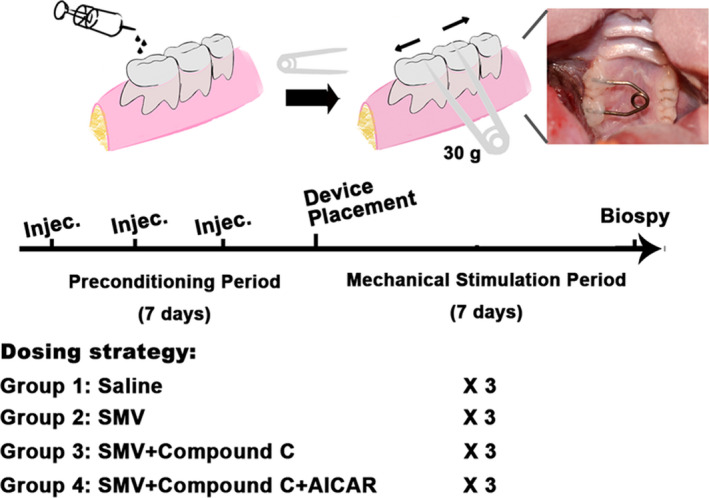
Study design and grouping strategy. Schematic diagram shows the steps of animal study and the grouping strategy depending on the drug administration method

In general, a total of 24 SD rats received orthodontic appliance (n = 24) placement. During the pre‐conditioning period of 7 days, all rats were in good general health and showed any adverse reactions to the injection procedures. No orthodontic appliance was lost in the mechanical stimulation period (Figure 5). After the appliance installation, sites were observed with limited inflammatory responses.

### 3.5 Micro‐CT assessment

**Figure 6:**
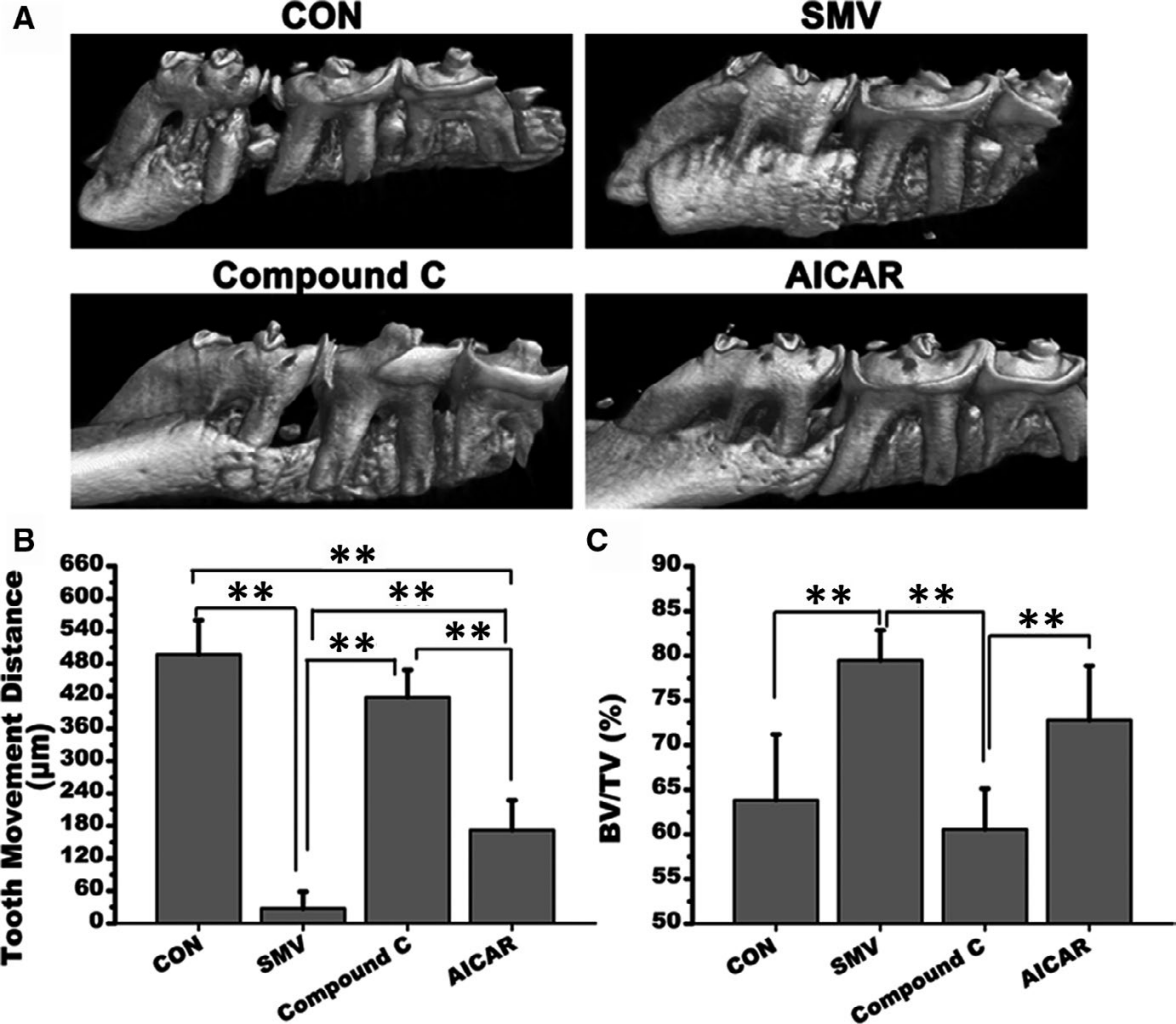
Micro‐CT detection. A, 3D reconstruction photos of the four groups. B, Tooth movement distance of the first maxillary molar after the 7‐day orthodontic treatment (μm). C, Histomorphometric findings of BV/TV (%; **, *P* < .01)

The maxillae samples were examined by micro‐CT before the following process. Massive bone resorption was noted around the distal root of the first maxillary molar in the control group, while the height of the bones around the distal roots in the SMV group was basically maintained at the bifurcation position (Figure 6A). The moved distance of first maxillary molar in control group was 497.13 ± 63.28 μm, which was significantly higher than the SMV group 27.56 ± 31.15 μm and AICAR group 172.45 ± 55.05 μm (**, *P* < .01). There was no statistical difference between the control group and the Compound C group 417.91 ± 50.36 μm (*P* > .05; Figure 6B). The BV/TV percentage was consistent with 3D reconstruction observation. The control group 63.84 ± 7.36% was the lowest, while the SMV group 79.49 ± 3.38% the maximum (**, *P* < .01). The treatment of Compound C reduced the BV/TV to 60.55 ± 4.56%, and the addition of AICAR significantly increased the ratio to 72.8 ± 6.08% (**, *P* < .01; Figure 6C).

### 3.6 Histological observation

**Figure 7:**
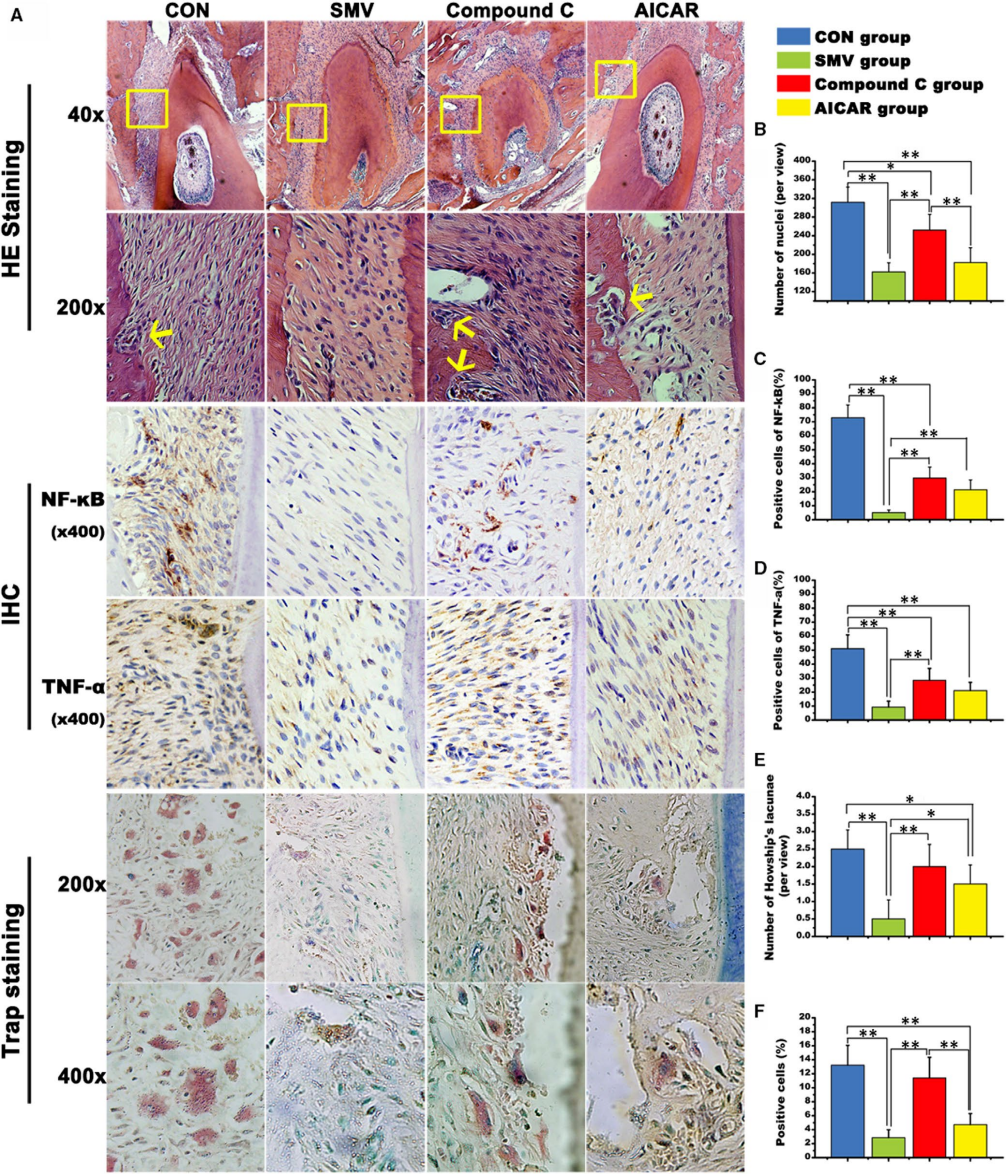
Histological and histomorphometric findings. A, H&E staining, immunohistological staining of NF‐κB and TNF‐ɑ, and Trap staining of the four groups. B, Number of cell nuclei; C, Positive cells assessment of NF‐κB (%); D, Positive cells assessment of TNF‐ɑ (%); E, Number of Howship's lacunae; F, Positive cell of Trap‐stained cells (%; *, *P* < .05; **, *P* < .01)

Rest specimens were decalcified for paraffin sections preparing. H&E staining showed that the pressure side of the periodontium was narrower than the tension side (Figure 7A). Observing the periodontal ligament on the tension side of the distal root (within the yellow square), we found that the thickness of periodontal ligament was larger in the control group, their arrangement was more chaotic, and the nucleus appeared to be significantly longer than the other three groups. Meanwhile, the change of periodontal space in the SMV group was limited. There was no obvious bone resorption site on the tension side, as indicated by the yellow arrows in the other three groups. Little influx of inflammatory cells was detected in the SMV group. The histological findings of Compound C group and AICAR group were between the former two groups. In terms of cell arrangement, the Compound C group was more similar to the control group. This trend was proved by the histomorphometric assessment of cell nuclei numbers (Figure 7B). Specifically, there were more cells in the control group (311.67 ± 32.58) than the other three groups, as 162.17 ± 19.86 for SMV group, 252 ± 34.02 for Compound C group, and 182.5 ± 31.46 for AICAR group.

The immunohistological staining showed that the NF‐κB and the TNF‐α expression was strongly positive on the tension side of the moved molar in the control group. These two factors were both weakest expressed in the SMV group. However, their uniformly scattered coloration could be seen in the periodontal ligament of the Compound C group. The stained cells percentage of the two factors demonstrated identical changes among the four groups, which was similar to the histomorphometric results of the H&E staining (Figure 7C,D). For Trap staining, multiple red‐stained osteoclasts were observed in the control group. In the SMV group, a characteristic bone resorption pit with red‐stained osteoclasts as well as a resorbed bone surface was hardly detected. Similarly, the stained Howship's lacuna was untypical in the AICAR group. The SMV group displayed the lower number (0.5 ± 0.55) of Howship's lacunae as compared to the control (2.5 ± 0.55) and Compound C group (2 ± 0.63) (**, *P* < .01) and the AICAR group (1.5 ± 0.55) (*, *P* < .05). There was also a statistic difference between the control group and the AICAR group (*, *P* < .05; Figure 7E). In terms of cell size and cell amount, the control group was obviously larger than the other three groups (**, *P* < .01; Figure 7F).

## 4 DISCUSSIONS

Control of stabilization during orthodontic treatment is considered of significant importance since it helps avoid undesirable tooth movements that may occur as a consequence of the reaction forces applied to move teeth.^39^ Pharmacological therapy of clinically accepted medicines is a shortcut to complement the orthodontic appliance which unavoidably leads to a higher level of pain and discomfort. Recent studies have found that SMV is effective in inhibition of relapse after orthodontic treatment. This study established an orthodontic tensile stressing model both in vitro and in vivo to analyse the molecular mechanism in SMV‐induced osteogenesis under biomechanical inflammatory condition. It is the first time to identify that the inhibition of AMPK/MAPK/NF‐κB pathway by local administrated SMV could enhance tooth anchorage in alveolar during OTM.

The periodontal ligament is a layer of connective tissue between the root of the tooth and the alveolar bone.^40^, ^41^ PDLCs contribute to the regeneration of periodontal ligament, cementum and alveolar bone ^42^, ^43^ and are recognized as the histological basis for OTM.^29^, ^44^, ^45^ However, previous studies explaining the bone‐specific anabolic property of SMV mostly based on observation of static‐culturing osteoblasts.^46^, ^47^ Researchers found that SMV could promote the expression of ALP and OCN from increased secretion of BMP‐2.^48^, ^49^ Concerning the cell origination for the metabolism of periodontium and alveolar during OTM, we applied human PDLCs for the in vitro tests. We found that the low concentration SMV could elevate the expression levels of BMP‐2 as well as VEGF in PDLCs under a tensile condition in vitro (Figures 1 and 3). This was consistent with previous findings that SMV could lift the expression of BMP‐2 and VEGF mRNA in osteoblasts after local application of SMV.^50^ Since bone formation is a coupling process involving osteogenesis and angiogenesis, this result proves the bone anabolic property of SMV on PDLCs in the tensile condition. Interestingly, there were significant differences among the gene expression levels of BMP‐2, VEGF, TNF‐ɑ and AMPK on most time‐points between the tension and the static groups, which highlighted the importance of tension force system application.

NF‐κB is a well‐known transcription factor associated with inflammation and bone resorption.^51^ Many studies reported orthodontic mechanical stress could trigger the NF‐κB pathway activation, which mediated the expression of pro‐inflammatory genes such as TNF‐α and IL‐6, as proved in our histological findings.^52^ Therefore, inhibition of NF‐κB activation is an important process in increasing tooth anchorage. On the other hand, ERK and p38 MAPK belong to the MAPKs signalling pathway, which played a central role involved in inflammatory response and cell survival.^53^, ^54^ For certain, recent studies indicated the phosphorylation of p38 and ERK1/2 MAPKS was activated in stress‐stimulated PDLCs.^55^ We then verified the effect of SMV on the MAPKs signalling pathway, and our results revealed SMV markedly inhibited stress‐stimulated ERK and p38 MAPK phosphorylation in PDLCs, and these manifestations had significant changes after treatment with Compound C, the inhibitor of AMPK. The pre‐condition of AICAR significantly reversed the inhibition trend and increased the pro‐inflammatory factors expression level. Furthermore, we tested the expression level of TNF‐α and IL‐1. Similar changes were identified under the Compound C and AICAR treatment. AMPK is an energy sensor that is activated by several types of stresses and has been described as a negative regulator of inflammatory response to IL‐1, IL‐6 and TNF‐α.^56^ Here, we detected the inhibition of NF‐κB phosphorylation and the most significant reduction in IL‐6 levels among the 20 cytokine in the SMV stimulated PDLCs (Figure 2). Thus, a relationship was figured between AMPK activation and SMV‐induced anti‐inflammation. Previous studies reported that the activation of AMPK can reduce the inflammatory level caused by overload exercise on muscle cells and reverse harmful inflammation activity to a state of regenerative inflammation that induces muscle regeneration.^57^ Studies also reported that AMPK signalling is a negative regulator of NF‐κB‐TNFα inflammatory axis in the treatment of hepatic inflammation and fibrosis.^58^ Our work confirmed with previously studies and first identified that the anti‐inflammatory and bone protection effect of SMV were associated with suppressing MAPKs phosphorylation and blocking NF‐κB activation via AMPK.

However, this study mainly discussed the tension irritation which could not represent the integral stressing status of the tooth under orthodontic force. Also, a complete understanding of mechanic bone formation requires the identification and analysis of osteoclast and its responses. After that, this study recognized the inhibition of AMPK/MAKP/NF‐κB pathway mediating SMV bone‐protective effect on the enhancement of tooth anchorage during OTM.

## CONFLICT OF INTEREST

The authors confirm that there are no known conflicts of interest associated with this publication.

## AUTHOR CONTRIBUTIONS

Xu Qin: involved in conceptualization, supervision and project administration. Babak Baban involved in methodology and wrote—reviewed and edited. Guangxun Zhu helped in software. Jing Mao validated the study. Lianyi Xu involved in formal analysis. Xiaojuan Sun and Lianyi Xu involved in investigation, resources and data curation. Guangxun Zhu and Jing Mao wrote and prepared the original draft. Lianyi Xu and Xu Qin acquired funding.

## Data Availability

The data that support the findings of this study are available from the corresponding author upon reasonable request.

## References

[1] Meikle MC . Remodeling the dentofacial skeleton: the biological basis of orthodontics and dentofacial orthopedics. J Dent Res. 2007;86:12‐24. 17189458 10.1177/154405910708600103

[2] Kommuri K , Javed F , Akram Z , Khan J . Effect of statins on orthodontic tooth movement: a systematic review of animal and clinical studies. Arch Oral Biol. 2020;111:104665. 31951846 10.1016/j.archoralbio.2020.104665

[3] Makrygiannakis MA , Kaklamanos EG , Athanasiou AE . Does common prescription medication affect the rate of orthodontic tooth movement? A systematic review. Eur J Orthod. 2018;40:649‐659. 29522172 10.1093/ejo/cjy001

[4] Santana LG , Duarte‐Rodrigues L , Alves‐Duarte AC , et al. Systematic review of biological therapy to accelerate orthodontic tooth movement in animals: translational approach. Arch Oral Biol. 2020;110:104597. 31739076 10.1016/j.archoralbio.2019.104597

[5] Jin J , Zhang X , Lu Z , et al. Simvastatin inhibits lipopolysaccharide‐induced osteoclastogenesis and reduces alveolar bone loss in experimental periodontal disease. J Periodontal Res. 2014;49(4):518‐526. 24117880 10.1111/jre.12132 PMC3979522

[6] Raafat SN , Amin RM , Elmazar MM , Khattab MM , El‐Khatib AS . The sole and combined effect of simvastatin and platelet rich fibrin as a filling material in induced bone defect in tibia of albino rats. Bone. 2018;117:60‐69. 30208342 10.1016/j.bone.2018.09.003

[7] Moshiri A , Sharifi AM , Oryan A . Role of Simvastatin on fracture healing and osteoporosis: a systematic review on in vivo investigations. Clin Exp Pharmacol Physiol. 2016;43:659‐684. 27061579 10.1111/1440-1681.12577

[8] Bing W , Pang X , Qu Q , et al. Simvastatin improves the homing of BMSCs via the PI3K/AKT/miR‐9 pathway. J Cell Mol Med. 2016;20:949‐961. 26871266 10.1111/jcmm.12795 PMC4831354

[9] Pradeep AR , Thorat MS . Clinical effect of subgingivally delivered simvastatin in the treatment of patients with chronic periodontitis: a randomized clinical trial. J Periodontol. 2010;81:214‐222. 20151799 10.1902/jop.2009.090429

[10] Surve SM , Acharya AB , Thakur SL . Efficacy of subgingivally delivered atorvastatin and simvastatin as an adjunct to scaling and root planing. Drug Metabol Personal Ther. 2015;30:263‐269. 26552068 10.1515/dmpt-2015-0024

[11] Pradeep AR , Rao NS , Bajaj P , Kumari M . Efficacy of subgingivally delivered simvastatin in the treatment of patients with type 2 diabetes and chronic periodontitis: a randomized double‐masked controlled clinical trial. J Periodontol. 2013;84:24‐31. 22420871 10.1902/jop.2012.110721

[12] Makrygiannakis MA , Kaklamanos EG , Athanasiou AE . Effects of systemic medication on root resorption associated with orthodontic tooth movement: a systematic review of animal studies. Eur J Orthod. 2019;41:346‐359. 29992228 10.1093/ejo/cjy048

[13] Jahanbin A , Abtahi M , Namdar P , et al. Evaluation of the effects of subgingival injection of Simvastatin on space re‐opening after orthodontic space closure in adults. J Dent Res Dent Clin Dent Prospects. 2016;10:3‐7. 27092208 10.15171/joddd.2016.001 PMC4831608

[14] Zhao P , Sun X , Chaggan C , et al. An AMPK‐caspase‐6 axis controls liver damage in nonalcoholic steatohepatitis. Science. 2020;367:652‐660. 32029622 10.1126/science.aay0542 PMC8012106

[15] Kim SH , Kim G , Han DH , et al. Ezetimibe ameliorates steatohepatitis via AMP activated protein kinase‐TFEB‐mediated activation of autophagy and NLRP3 inflammasome inhibition. Autophagy. 2017;13:1767‐1781. 28933629 10.1080/15548627.2017.1356977 PMC5640190

[16] Mihaylova MM , Shaw RJ . The AMPK signalling pathway coordinates cell growth, autophagy and metabolism. Nat Cell Biol. 2011;13:1016‐1023. 21892142 10.1038/ncb2329 PMC3249400

[17] Ghanim H , Green K , Abuaysheh S , et al. Ezetimibe and simvastatin combination inhibits and reverses the pro‐inflammatory and pro‐atherogenic effects of cream in obese patients. Atherosclerosis. 2017;263:278‐286. 28711708 10.1016/j.atherosclerosis.2017.06.010

[18] Maneechotesuwan K , Wongkajornsilp A , Adcock IM , Barnes PJ . Simvastatin suppresses airway IL‐17 and upregulates IL‐10 in patients with stable COPD. Chest. 2015;148:1164‐1176. 26043025 10.1378/chest.14-3138 PMC4631035

[19] Ji RC , Eshita Y , Kobayashi T , Hidano S , Kamiyama N , Onishi Y . Role of simvastatin in tumor lymphangiogenesis and lymph node metastasis. Clin Exp Metastasis. 2018;35:785‐796. 30255290 10.1007/s10585-018-9940-8

[20] Lee J , Jung KH , Park YS , et al. Simvastatin plus irinotecan, 5‐fluorouracil, and leucovorin (FOLFIRI) as first‐line chemotherapy in metastatic colorectal patients: a multicenter phase II study. Cancer Chemother Pharmacol. 2009;64:657‐663. 19169686 10.1007/s00280-008-0913-5

[21] de Oliveira DM , de Oliveira EM , Ferrari Mde F , et al. Simvastatin ameliorates experimental autoimmune encephalomyelitis by inhibiting Th1/Th17 response and cellular infiltration. Inflammopharmacology. 2015;23:343‐354. 26559850 10.1007/s10787-015-0252-1

[22] Agarwal P , Rashighi M , Essien KI , et al. Simvastatin prevents and reverses depigmentation in a mouse model of vitiligo. J Invest Dermatol. 2015;135:1080‐1088. 25521459 10.1038/jid.2014.529 PMC4366328

[23] Qin X , Hoda MN , Susin C , et al. Increased innate lymphoid cells in periodontal tissue of the murine model of periodontitis: the role of AMP‐activated protein kinase and relevance for the human condition. Front Immunol. 2017;8:922. 28861078 10.3389/fimmu.2017.00922 PMC5559469

[24] Wang S , Kim M , Ali Z , Ong K , Pae EK , Chung MK . TRPV1 and TRPV1‐expressing nociceptors mediate orofacial pain behaviors in a mouse model of orthodontic tooth movement. Front Physiol. 2019;10:1207. 31620023 10.3389/fphys.2019.01207 PMC6763553

[25] Xia LG , Zhang ZY , Chen L , et al. Proliferation and osteogenic differentiation of human periodontal ligament cells on akermanite and beta‐Tcp bioceramics. Eur Cells Mater. 2011;22:68‐83. 10.22203/ecm.v022a06 21761393

[26] Liu J , Wang X , Zheng M , Luan Q . Lipopolysaccharide from Porphyromonas gingivalis promotes autophagy of human gingival fibroblasts through the PI3K/Akt/mTOR signaling pathway. Life Sci. 2018;211:133‐139. 30218719 10.1016/j.lfs.2018.09.023

[27] Xu L , Sun X , Cao K , et al. Hypoxia induces osteogenesis in rabbit adipose‐derived stem cells overexpressing bone morphogenic protein‐2. Oral Dis. 2014;20(5):430‐439. 23865899 10.1111/odi.12148

[28] Yamada M , Watanabe J , Ueno T , Ogawa T , Egusa H . Cytoprotective preconditioning of osteoblast‐like cells with N‐acetyl‐L‐cysteine for bone regeneration in cell therapy. Int J Mol Sci. 2019;20:5199. 10.3390/ijms20205199 PMC6834301 31635184

[29] Wu Y , Ou Y , Liao C , Liang S , Wang Y . High‐throughput sequencing analysis of the expression profile of microRNAs and target genes in mechanical force‐induced osteoblastic/cementoblastic differentiation of human periodontal ligament cells. Am J Transl Res. 2019;11:3398‐3411. 31312353 PMC6614645

[30] Ke F , Zhang L , Liu Z , et al. Autocrine interleukin‐6 drives skin‐derived mesenchymal stem cell trafficking via regulating voltage‐gated Ca(2+) channels. Stem Cells. 2014;32:2799‐2810. 24906203 10.1002/stem.1763

[31] Wang XF , Zhang JY , Li L , Zhao XY , Tao HL , Zhang L . Metformin improves cardiac function in rats via activation of AMP‐activated protein kinase. Clin Exp Pharmacol Physiol. 2011;38:94‐101. 21143620 10.1111/j.1440-1681.2010.05470.x

[32] Yozgatian JH , Zeredo JL , Hotokezaka H , Koga Y , Toda K , Yoshida N . Emotional stress‐ and pain‐related behaviors evoked by experimental tooth movement. Angle Orthod. 2008;78:487‐494. 18416621 10.2319/040207-165.1

[33] Takamori Y , Atsuta I , Nakamura H , Sawase T , Koyano K , Hara Y . Histopathological comparison of the onset of peri‐implantitis and periodontitis in rats. Clin Oral Implants Res. 2017;28:163‐170. 26804139 10.1111/clr.12777

[34] Wang B , Wu Y , Yu H , Jiang L , Fang B , Guo Q . The effects of NELL on corticotomy‐assisted tooth movement and osteogenesis in a rat model. Biomed Mater Eng. 2018;29:757‐771. 30282332 10.3233/BME-181021

[35] Klein Y , Fleissig O , Stabholz A , Chaushu S , Polak D . Bone regeneration with bovine bone impairs orthodontic tooth movement despite proper osseous wound healing in a novel mouse model. J Periodontol. 2019;90:189‐199. 30059146 10.1002/JPER.17-0550

[36] An J , Li Y , Liu Z , Wang R , Zhang B . A micro‐CT study of microstructure change of alveolar bone during orthodontic tooth movement under different force magnitudes in rats. Exp Ther Med. 2017;13:1793‐1798. 28565769 10.3892/etm.2017.4186 PMC5443305

[37] Qin X , Zou F , Chen W , et al. Demineralized dentin as a semi‐rigid barrier for guiding periodontal tissue regeneration. J Periodontol. 2015;86:1370‐1379. 26317665 10.1902/jop.2015.150271

[38] Lee YC , Chan YH , Hsieh SC , Lew WZ , Feng SW . Comparing the osteogenic potentials and bone regeneration capacities of bone marrow and dental pulp mesenchymal stem cells in a rabbit calvarial bone defect model. Int J Mol Sci. 2019;20:5015. 10.3390/ijms20205015 PMC6834129 31658685

[39] Papadopoulos MA , Papageorgiou SN , Zogakis IP . Clinical effectiveness of orthodontic miniscrew implants: a meta‐analysis. J Dent Res. 2011;90:969‐976. 21593250 10.1177/0022034511409236

[40] Seo BM , Miura M , Gronthos S , et al. Investigation of multipotent postnatal stem cells from human periodontal ligament. Lancet. 2004;364:149‐155. 15246727 10.1016/S0140-6736(04)16627-0

[41] Li X , Liao D , Sun G , Chu H . Odontogenesis and neuronal differentiation characteristics of periodontal ligament stem cells from beagle dog. J Cell Mol Med. 2020;24(9):5146‐5151. 32202359 10.1111/jcmm.15158 PMC7205787

[42] Tsumanuma Y , Iwata T , Washio K , et al. Comparison of different tissue‐derived stem cell sheets for periodontal regeneration in a canine 1‐wall defect model. Biomaterials. 2011;32:5819‐5825. 21605900 10.1016/j.biomaterials.2011.04.071

[43] Lei M , Li K , Li B , Gao LN , Chen FM , Jin Y . Mesenchymal stem cell characteristics of dental pulp and periodontal ligament stem cells after in vivo transplantation. Biomaterials. 2014;35:6332‐6343. 24824581 10.1016/j.biomaterials.2014.04.071

[44] Li S , Li Q , Zhu Y , Hu W . GDF15 induced by compressive force contributes to osteoclast differentiation in human periodontal ligament cells. Exp Cell Res. 2020;387:111745. 31765611 10.1016/j.yexcr.2019.111745

[45] Frank D , Cser A , Kolarovszki B , Farkas N , Miseta A , Nagy T . Mechanical stress alters protein O‐GlcNAc in human periodontal ligament cells. J Cell Mol Med. 2019;23:6251‐6259. 31237748 10.1111/jcmm.14509 PMC6714205

[46] Mundy G , Garrett R , Harris S , et al. Stimulation of bone formation in vitro and in rodents by statins. Science. 1999;286:1946‐1949. 10583956 10.1126/science.286.5446.1946

[47] Tan J , Yang N , Fu X , et al. Single‐dose local simvastatin injection improves implant fixation via increased angiogenesis and bone formation in an ovariectomized rat model. Med Sci Monit. 2015;21:1428‐1439. 25982481 10.12659/MSM.892247 PMC4448596

[48] Yaghobee S , Panjnoush M , Rafiei SC , et al. Effect of simvastatin on bone regeneration: a histologic and histomorphometric analysis. J Oral Maxillofac Surg. 2020;78(6):927‐934. 32084353 10.1016/j.joms.2020.01.016

[49] Feng C , Xiao L , Yu JC , et al. Simvastatin promotes osteogenic differentiation of mesenchymal stem cells in rat model of osteoporosis through BMP‐2/Smads signaling pathway. Eur Rev Med Pharmacol Sci. 2020;24:434‐443. 31957858 10.26355/eurrev_202001_19943

[50] Liu C , Wu Z , Sun HC . The effect of simvastatin on mRNA expression of transforming growth factor‐beta1, bone morphogenetic protein‐2 and vascular endothelial growth factor in tooth extraction socket. Int J Oral Sci. 2009;1:90‐98. 20687301 10.4248/ijos.08011 PMC3735797

[51] Fan X , He L , Dai Q , et al. Interleukin‐1beta augments the angiogenesis of endothelial progenitor cells in an NF‐kappaB/CXCR7‐dependent manner. J Cell Mol Med. 2020;24:5605–5614. 32239650 10.1111/jcmm.15220 PMC7214148

[52] Dong M , Yu X , Chen W , et al. Osteopontin promotes bone destruction in periapical periodontitis by activating the NF‐kappaB pathway. Cell Physiol Biochem. 2018;49:884‐898. 30184545 10.1159/000493219

[53] Wu H , Zhao G , Jiang K , et al. Plantamajoside ameliorates lipopolysaccharide‐induced acute lung injury via suppressing NF‐kappaB and MAPK activation. Int Immunopharmacol. 2016;35:315‐322. 27089391 10.1016/j.intimp.2016.04.013

[54] Huang SW , Chyuan IT , Shiue C , Yu MC , Hsu YF , Hsu MJ . Lovastatin‐mediated MCF‐7 cancer cell death involves LKB1‐AMPK‐p38MAPK‐p53‐survivin signalling cascade. J Cell Mol Med. 2020;24:1822‐1836. 31821701 10.1111/jcmm.14879 PMC6991643

[55] Jiang L , Tang Z . Expression and regulation of the ERK1/2 and p38 MAPK signaling pathways in periodontal tissue remodeling of orthodontic tooth movement. Mol Med Rep. 2018;17:1499‐1506. 29138812 10.3892/mmr.2017.8021 PMC5780090

[56] Jeon SM , Chandel NS , Hay N . AMPK regulates NADPH homeostasis to promote tumour cell survival during energy stress. Nature. 2012;485:661‐665. 22660331 10.1038/nature11066 PMC3607316

[57] Saito Y , Chikenji TS , Matsumura T , Nakano M , Fujimiya M . Exercise enhances skeletal muscle regeneration by promoting senescence in fibro‐adipogenic progenitors. Nat Commun. 2020;11:889. 32060352 10.1038/s41467-020-14734-x PMC7021787

[58] Zhang T , Hu J , Wang X , et al. MicroRNA‐378 promotes hepatic inflammation and fibrosis via modulation of the NF‐kappaB‐TNFalpha pathway. J Hepatol. 2019;70:87‐96. 30218679 10.1016/j.jhep.2018.08.026 PMC6554744

